# Mycotoxin Research in Algeria: A Comprehensive Review of Trends, Challenges, and Future Directions

**DOI:** 10.3390/toxins17100492

**Published:** 2025-10-03

**Authors:** Yamina Ben Miri, Imene Chentir, Aldjia Taoudiat, Amina Benabdallah, Marta Herrera

**Affiliations:** 1 Department of Biochemistry and Microbiology, Faculty of Sciences, Mohamed Boudiaf University, M’sila 28000, Algeria; yamina.benmiri@univ-msila.dz; 2 Higher School of Food Science and Agri-Food Industry, Laboratory of Food, Processing, Control and Agri-Resources Valorization, Algiers 16200, Algeria; i.chentir@essaia.dz; 3 Department of Agronomy, Faculty of Life and Nature Sciences, Akli Mohand Oulhadj University, Bouira 10000, Algeria; taoudiatnaima@gmail.com; 4 Laboratory on Biodiversity and Ecosystem Pollution, Faculty of Life and Nature Sciences, University Chadli Bendjedid, El-Tarf 36000, Algeria; benabdallah-amina@univ-eltarf.dz; 5 Instituto Agroalimentario de Aragón—IA2, Facultad de Veterinaria, Universidad de Zaragoza-CITA, 50013 Zaragoza, Spain

**Keywords:** mycotoxin, Algeria, challenges, food safety, public health, collaborative efforts

## Abstract

This review offers a comprehensive overview of mycotoxin research and related publications in Algeria, outlining key trends, existing challenges, and prospects for future advancement. Despite limited exploration of mycotoxins in Algeria, researchers have made significant progress in understanding mycotoxin contamination and its effects on food safety and public health. The review delves into both research achievements and the challenges encountered in resource-limited settings, while also exploring strategies employed to surmount these obstacles. Through an analysis of existing literature, key themes emerge regarding mycotoxin identification, detection methods, mitigation strategies, and their implications. The importance of collaborative efforts among academia and government agencies is underscored as pivotal in addressing mycotoxin-related challenges. Moreover, this review identified gaps in current research and offered recommendations for future investigations, aiming to advance mycotoxin research in Algeria and beyond.

## 1. Introduction

Losses in food supplies during pre-harvest and post-harvest stages can result from various factors, including both biotic and abiotic influences. In regions with tropical climates that are hot and humid, the deterioration of stored food due to fungal activity remains a persistent challenge. Harvested grains are particularly vulnerable to contamination by a range of fungi genera, including *Aspergillus*, *Alternaria*, *Fusarium*, *Cladosporium*, *Penicillium*, *Mucor*, and *Rhizopus* [1]. Under specific conditions, certain fungi, including those from the *Aspergillus*, *Fusarium*, *Penicillium*, and *Alternaria* genera, can produce toxic secondary metabolites known as mycotoxins [2,3]. These compounds have the potential to contaminate both food and animal feed, representing a serious threat to human and animal health. Among the most studied mycotoxins are aflatoxins (AFs), ochratoxin A (OTA), patulin (PAT), fumonisins (FMs), deoxynivalenol (DON), and zearalenone (ZEN) [4,5]. This contamination not only threatens agricultural productivity but also poses significant health risks to consumers worldwide [6].

It is concerning that most African countries have not updated mycotoxin regulations in recent years. Even where regulatory frameworks exist, they often apply only to products intended for export, leaving domestic markets insufficiently protected [7]. Morocco is one of the few African countries with a comprehensive regulatory system, closely aligned with European Union standards. These regulations cover mycotoxins such as AFs, OTA, FMs, DON, PAT, and ZEN in a wide range of primary agricultural products and foodstuffs [8]. Moroccan authorities have adopted new regulations to set maximum regulatory limits (MLs) for certain mycotoxins in foodstuffs. These regulations were recently repealed and modified by the annex of Joint Decree n°2410-22 of 14 September 2022 (Bulletin Officiel, 2022) [8]. In contrast, Algeria and many other African countries regulate only aflatoxins, despite the demonstrated presence of other mycotoxins in food. Algerian regulatory limits for aflatoxin B1 (AFB1) and total aflatoxins (AFs: the sum of AFB1, AFB2, AFG1, and AFG2) have been set at 10 and 20 μg/kg, respectively, in peanuts, nuts, and cereals. These limits, however, are often more rigorously applied to export commodities, while products destined for local markets may be subject to less stringent control, potentially increasing the risk of exposure to food products that do not fully comply with safety standards. Until now, Algeria has not established maximum levels for OTA, DON, and ZEN in foodstuffs [9].

Algeria emerges as a distinctive focal point, shaped by its diverse agricultural heritage, climatic variations, and cultural richness. From the verdant oases of the Sahara to the fertile valleys of the Tell Atlas Mountains, the country’s geographical diversity supports both agricultural abundance and conditions favourable to mycotoxin production. Furthermore, the country’s vibrant cultural mosaic, interwoven with Arab and Mediterranean traditions, adds layers of complexity to the intricate relationship between agricultural practices, food production, and mycotoxin exposure [9,10,11].

Algeria is a North African country whose climate is characterised by high temperatures and high relative humidity in some areas that seems to stimulate mycotoxigenic moulds growth and toxinogenesis, in which cereals and cereal-based products represent a staple food for the population [12].

Currently, given that a substantial portion of cereals sold in Algeria is imported and there is limited information on toxin contamination, various preventive measures have been implemented to address health risks associated with mycotoxin exposure. These measures include the enforcement of legislation, the adoption of good agricultural practices, and the monitoring of contamination levels. However, it is crucial to gather evidence and data on the presence and levels of mycotoxins to manage risks effectively. Accurate data on mycotoxin exposure is essential for risk assessment, management efforts, and the development of appropriate legislation for the monitoring and control of mycotoxin contamination in food [13].

Additionally, over the past three decades, the effects of climate change have become increasingly evident. In the context of Algeria, this phenomenon is marked by rising average temperatures and greater variability in rainfall patterns, both of which may influence fungal growth and mycotoxin production [14]. These environmental shifts, along with socioeconomic and regulatory factors, contribute to shape the landscape of research dedicated to understanding and mitigating the impact of mycotoxins within the country [15].

A comprehensive exploration of mycotoxin research and publications in Algeria is essential to gain insights into the country’s specific challenges and opportunities in addressing these contaminants.

In summary, Algeria’s climatic diversity, ranging from Mediterranean coastal areas to arid desert regions, creates conditions that favor fungal growth and mycotoxin production. Together with the high dietary reliance on cereals and cereal-based traditional foods such as couscous and bread, these factors contribute significantly to the risk of mycotoxin exposure in the country.

This review conducts an in-depth examination of mycotoxin-related research in Algeria to map the current state of knowledge regarding contamination in the national food systems. It involves examining existing literature to discern prevalent trends, assess methodologies, and pinpoint areas where further investigation is needed. Furthermore, this review represents the first comprehensive review dedicated exclusively to mycotoxin research in Algeria.

Synthesizing insights from diverse academic studies, this review seeks to provide a nuanced understanding of the complex factors contributing to mycotoxin occurrence and exposure in Algeria. This synthesis identifies pathways for targeted intervention and innovative solutions to address the challenges associated with mycotoxin contamination in Algerian food systems. Ultimately, the goal is to enhance food safety and public health, both within Algeria and in international contexts. Leveraging the insights from this comprehensive review, stakeholders (including policymakers, researchers and industry actors) can collaborate to mitigate mycotoxin risks, safeguard consumer well-being, and promote resilient food systems that benefit Algerian communities and beyond.

This review addresses several objectives: assessing research progress, exploring obstacles and resource constraints, and evaluating the state of regulatory and scientific efforts within Algeria. Despite the increasing recognition of mycotoxin contamination as a significant issue, the field remains largely unexplored due to the limited number of studies and research initiatives. Through this analysis, this review aims to identify challenges such as deficiencies and regulatory gaps in Algeria, laying the groundwork for targeted interventions and capacity-building initiatives.

## 2. Research Methodology

**Figure 1:**
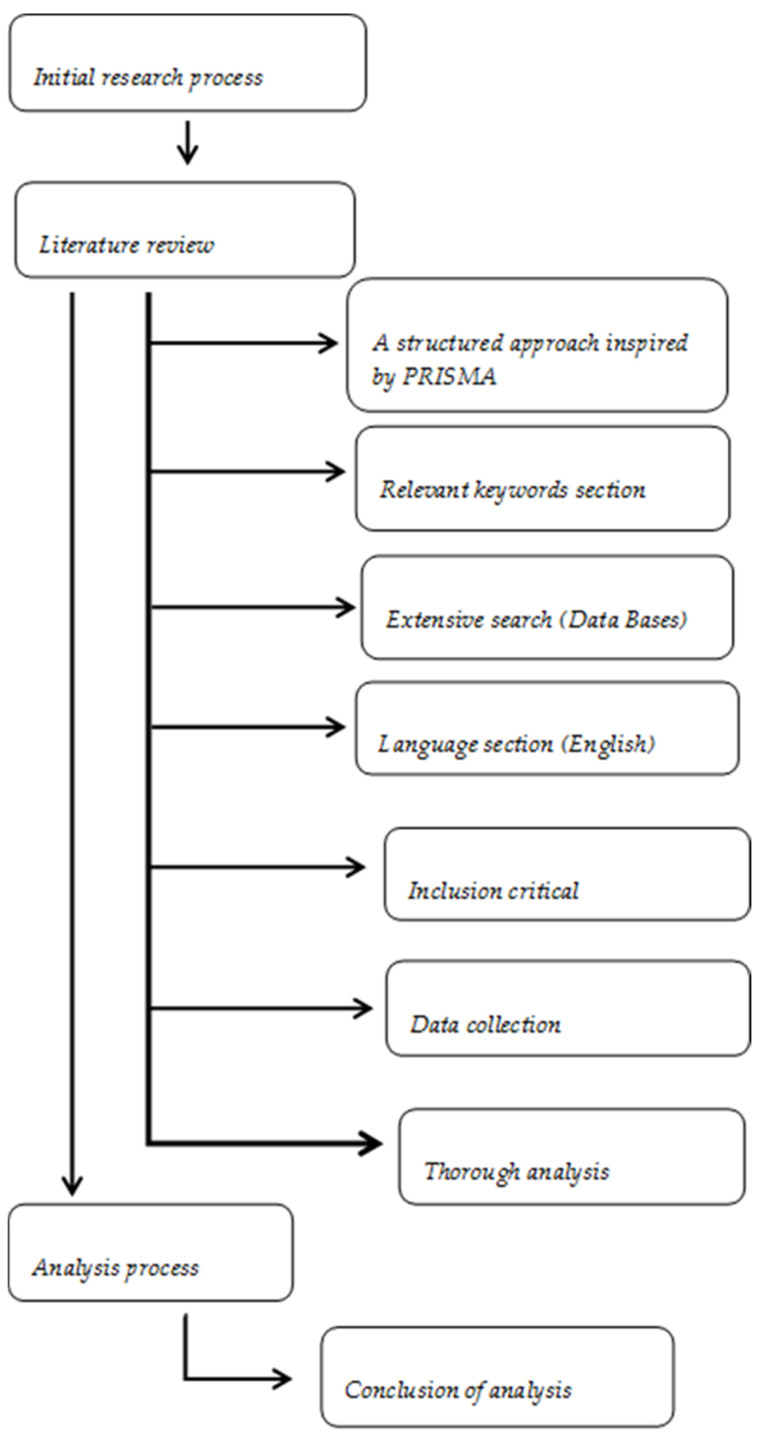
Structured research methodology inspired by PRISMA guidelines.

Inspired by the Preferred Reporting Items for Systematic reviews and Meta-Analyses (PRISMA) guidelines, this review followed a structured methodology starting with a carefully designed search strategy (Figure 1). Relevant keywords, such as mycotoxin, Algeria, food, agricultural practices, mitigation, detection, health implications, and regulatory measures, were selected to ensure broad and representative coverage of the topic. Comprehensive literature searches were conducted across major scientific databases, including PubMed, Scopus, and Web of Science. Publications available in English or French were prioritized to maximize relevance within the Algerian context.

Following the search phase, data extraction was carried out systematically. Information was collected on multiple variables, including: (i) mycotoxin occurrence, prevalence, and levels in Algerian agricultural products; (ii) analytical approaches used for detection, such as sampling strategies, analytical techniques and validation procedures; (iii) health implications of mycotoxin exposure in Algerian populations, supported by clinical and epidemiological findings; (iv) regulatory aspects, including existing legal thresholds, monitoring systems, and enforcement practices; (v) bibliometric data such as publication year, journal, author affiliations, and citation metrics; (vi) collaboration networks between national and international research groups; (vii) funding sources supporting mycotoxin-related research in Algeria and (viii) emerging trends and research gaps identified in the scientific literature. Subsequently, the collected articles were then subjected to in-depth analysis to identify prevailing types of mycotoxins, commonly employed methodologies, geographical focus of the studies, collaboration patterns, and prospective research directions. This analytical process formed the basis for synthesizing insights and drawing conclusions in subsequent sections of the study.

## 3. Evolution of Mycotoxin Research in Algeria

Mycotoxin contamination poses a substantial challenge to worldwide food safety and public health, affecting agricultural economies, trade, and consumer well-being. In Algeria, characterized by diverse agricultural production and a reliance on staple crops, mycotoxin contamination has emerged as a critical concern due to its implications for both domestic consumption and food safety trade requirements.

**Figure 2:**
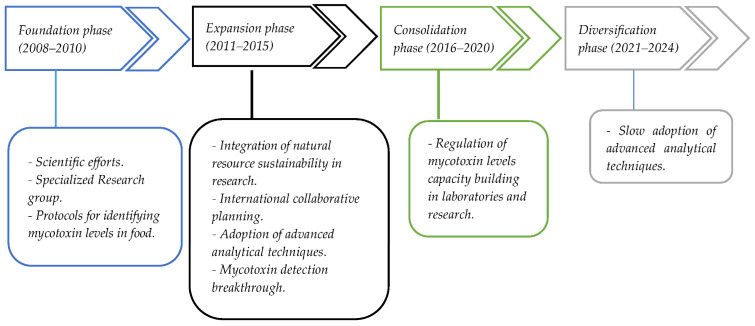
Key milestones and developments in mycotoxin Research in Algeria over the years.

In recent years, Algerian researchers have increasingly focused on understanding, reducing, and managing mycotoxin contamination in agricultural commodities, particularly staple crops such as cereals and nuts. The evolution of the national response to mycotoxin contamination can be categorized into four distinct phases (Figure 2): (i) Foundation phase (2008–2010), (ii) Expansion phase (2011–2015), (iii) Consolidation phase (2016–2020), and (iv) Diversification phase (2021–2024).

This review aims to trace the trajectory of mycotoxin-related research in Algeria, highlighting its milestones, challenges, and future measures. By examining the historical context and scientific advancements, this section provides insights into national efforts to mitigate mycotoxin risks and safeguard the safety of Algerian agricultural products.

### 3.1. Foundation Phase (2008–2010)

The foundation phase marks a crucial period in Algeria’s scientific efforts to control mycotoxin contamination in agricultural production. During this period, researchers began to recognize the harmful effects of mycotoxins on food safety and public health, particularly in staple crops such as wheat. This growing awareness led to the establishment of specialized research groups within academic institutions, specifically focused on challenges related to mycotoxins in Algerian agricultural commodities.

Initial efforts were addressed at establishing reliable protocols for the identification and quantification of mycotoxins in food matrices [16]. Techniques such as chromatography and immunoassays were introduced and adapted to local conditions, laying the groundwork for future studies. These developments provided a robust methodological foundation, enabling the systematic investigation of contamination pathways and facilitating the gradual development of national food safety measures.

A landmark development during this phase was the establishment of the first dedicated research team on mycotoxins within a national microbiology laboratory in Algiers [17,18]. This group brought valuable expertise in microbiology and molecular biology to the emerging field of mycotoxin research in Algeria. Its academic environment fostered interdisciplinary collaboration, drawing on knowledge from agriculture, food chemistry, and toxicology. The laboratory also initiated international collaborations with research institutes and universities abroad. These collaborations contributed to the exchange of methodologies and strengthened the analytical capacity for mycotoxin detection and management.

The pioneering work of Riba et al. [17,18] laid the scientific foundation for future research on mycotoxins in Algeria. Their studies focused on the occurrence and contamination levels of OTA and AFs in Algerian agricultural products and the factors contributing to this contamination, especially in wheat. Using systematic sampling across various stages of the production chain and adapting extraction techniques to wheat, they achieved accurate quantification of contamination levels and provided essential data for risk assessment. In 2008, the group employed HPLC with fluorescence detection (HPLC-FLD) to measure OTA production by *Aspergillus* and *Penicillium* isolates [17]. By 2010, they had extended their work to include the study of *Aspergillus* section *Flavi* and chromatographic determination of AFB1 in wheat samples [18].

These studies revealed the frequent occurrence of aflatoxigenic and ochratoxigenic fungal strains in Algerian wheat and underscored the need for stringent control during postharvest handling and storage. Riba et al. highlighted the significant role that inadequate storage conditions can play in mycotoxin accumulation and called for further research to better understand the population’s exposure to AFs and OTA. Their findings catalyzed the development of future research and policy actions aimed at mycotoxin mitigation and food safety improvement in Algeria.

Building on these initial efforts, the subsequent expansion phase (2011–2015) was characterized by a broader scope of investigations and the adoption of more analytical methods, which significantly enhanced national capacity to detect and assess mycotoxin contamination.

### 3.2. Expansion Phase (2011–2015)

This period was marked by a significant increase in research activity in Algeria, focusing on mycotoxin-related challenges through collaborative and interdisciplinary approaches. Researchers focused on their understanding of the interactions between fungi, crops, and environmental factors, aiding the development of effective intervention strategies. The adoption of advanced analytical techniques, such as liquid chromatography-mass spectrometry (LC-MS) and enzyme-linked immunosorbent assay (ELISA), significantly enhanced the precision and sensitivity of mycotoxin detection, thereby improving risk assessment and management efforts.

In this context, Yang et al. [19] investigated the pressing issue of internal fruit rot in sweet peppers caused by *Fusarium* species, identifying the production of mycotoxins such as beauvericin (BEA), fumonisin B1, and moniliformin (MON). Their findings underscored the importance of understanding mycotoxin contamination in agricultural products and its implications for food safety. Similarly, Guezlane-Tebibel et al. [20] focused on the natural mycobiota present in Chinese peanuts sold in Algiers, assessing the incidence of aflatoxigenic *Aspergillus* section *Flavi* species. Through advanced analytical techniques like HPLC, they detected AFs in peanut samples, emphasizing the need for ongoing monitoring and regulatory measures to ensure food safety. In another significant study led by Riba et al. [21], AF contamination in various nuts sampled from the Algerian market was investigated using HPLC. In 2015, Redouane-Salah et al. [22] conducted a study on the presence of AFM1 in milk consumed in Algeria, using immunoaffinity purification and liquid chromatography-fluorescence detection (LC-FLD). Their findings underscored the significance of detecting AFM1 contamination in dairy products.

During the same period, Yekkour et al. [23] isolated twelve *Fusarium culmorum* strains from infected barley and wheat roots in north Algeria. These isolates exhibited variable trichothecene production profiles, with two strains (Fc1 and Fc12) showing the highest deoxynivalenol (DON) levels, as confirmed by molecular characterization and HPLC. In the same year, Yekkour et al. [24] investigated the effects of DON produced by *F. culmorum* and *F. graminearum* on both plants and animals. Their study focused on understanding how DON triggers programmed cell death (PCD) in *Nicotiana tabacum* BY2 cells, revealing molecular mechanisms underlying this process. Their research revealed interconnected pathways triggered by DON. These included ROS generation linked to mitochondrial dysfunction and the transcriptional down-regulation of the alternative oxidase (Aox1) gene. Together, these findings indicate mitochondrial involvement in PCD induction. Additionally, DON was found to regulate ion channel activities, contributing to cell shrinkage, a characteristic of PCD. This study illuminated the complexity of DON-induced PCD in plant cells and provided insights into the intricate interplay between molecular pathways involved in this process, advancing the understanding of how mycotoxins impact cellular processes in plants, with implications for agriculture and food safety.

Although there were not numerous studies conducted during this phase, those carried out during this period have demonstrated a notable adoption of advanced analytical methods and technologies to delve deeper into the complexities of mycotoxin contamination. These studies represent a pioneering effort to use state-of-the-art methodologies in mycotoxin research, despite the relatively limited number of investigations conducted during this timeframe. This phase also marked the integration of advanced techniques, including HPLC-FLD and PCR assays targeting genes involved in trichothecene biosynthesis (ITS1-5.8S-ITS2 rDNA regions).

These approaches significantly improved sensitivity, precision, and specificity, enabling more accurate detection, quantification, and characterization of mycotoxins and their producing fungi. Overall, although limited in number, these studies expanded the scope of Algerian mycotoxin research beyond wheat to include nuts, milk, and horticultural crops, while introducing more sophisticated analytical methods. These methodological advances and diversified studies laid the groundwork for the subsequent consolidation phase (2016–2020), during which research activity became more structured and strategically aligned with national priorities.

### 3.3. Consolidation Phase (2016-2020)

The period from 2016 to 2020 represented a turning point in Algerian mycotoxin research, marked by the consolidation of earlier efforts into more structured and coordinated initiatives. Unlike the preceding expansion phase, which primarily broadened the scope of commodities studied, this stage was characterized by the establishment of regulatory frameworks and the strengthening of institutional capacity. Research activity not only increased but also became more strategically aligned with national food safety priorities.

Key achievements included the development of regulations for mycotoxin levels in agricultural products and sustained collaboration with industry stakeholders. These partnerships facilitated the exchange of knowledge and technologies, contributing to practical management strategies for contamination [9]. Capacity building also advanced through targeted training programs and scholarships supported by the Algerian Ministry of Higher Education and Scientific Research, which enabled researchers to pursue advanced studies despite resource constraints. Additional support from international funding agencies, including the Spanish State Research Agency and the Government of Aragon (Analysis and Food Safety Group), reinforced the national research infrastructure.

Within this framework, research directions expanded along several fronts. Surveillance and analytical advances contributed to more reliable detection and quantification of mycotoxins. Lahoum et al. [25] used HPLC coupled with a fluorescence detector for the extraction and quantification of AFB1 in food products, while Ait Mimoune et al. [26] applied HPLC-FLD for the determination of AF levels in peanuts, almonds, and dried figs. Zebiri et al. [9] investigated OTA in wheat and wheat-derived products using OtaCLEAN™ SMART immunoaffinity columns and HPLC-FLD, providing reliable quantification across multiple regions.

At the same time, crop-specific pathogen and mycotoxin investigations deepened knowledge of *Fusarium* and *Aspergillus* species. Azzoune et al. [27] confirmed the aflatoxigenic capacity of *Aspergillus* section Flavi in spices, Touati-Hattab et al. [28] identified *F. culmorum* as the primary pathogen responsible for *Fusarium* Head Blight and *Fusarium* root rot in Algerian wheat, Bouras et al. [29] studied the influence of nitrogen sources on Pyrenophora tritici-repentis growth and toxin production, Hadjout et al. [30] evaluated resistant breeding lines for FHB, Laraba et al. [31] applied molecular techniques for *Fusarium culmorum* characterization, and Djaaboub et al. [32] reported heavy *Fusarium* contamination in local and imported wheat with DON levels exceeding legal limits.

In parallel, natural antifungal strategies began to emerge, with Ben Miri et al. [33] testing Citrus essential oils against *A. flavus*, Ben Miri and Djenane [34] studying *Thymus capitatus* EO, and Belasli et al. [35] demonstrating laurel EO efficacy against A. flavus and AFB1 production.

Additional works expanded the knowledge base: Mazrou et al. [36] assessed OTA in grapes, Mahdjoubi et al. [37] employed UHPLC-MS/MS to quantify 15 mycotoxins in cereals, Mohammedi-Ameur et al. [38] detected AFM1 in raw milk samples, Bouti et al. [39] characterized *Aspergillus* section Flavi from animal feed, Krouma et al. [40] confirmed patulin production by *P. expansum* in apples, and Khaldi et al. [41] investigated infant flours, identifying high contamination and potential for control with *Anethum graveolens* extracts.

Overall, the distinctive feature of this phase was its focus on institutional strengthening, regulation, and initial exploration of natural inhibitors, which collectively provided a stable foundation for the subsequent diversification of research. This institutional consolidation paved the way for a diversification of research topics and methodologies in the following years (2021–2024).

### 3.4. Diversification Phase (2021–2024)

Between 2021 and 2024, mycotoxin research in Algeria showed diversification into new food and feed matrices, while also expanding investigations into natural mitigation approaches. However, unlike in other regions where innovation accelerated, Algerian research remained slower to adopt cutting-edge technologies such as biosensors, nanotechnology, or advanced data analytics. Traditional detection methods continued to dominate, and although natural and industrial control strategies gained visibility, the integration of novel detection tools was limited.

Within this context, research evolved along distinct thematic areas. The diversification of commodities studied became evident as Moussaoui et al. [42] investigated toxigenic moulds and mycotoxins in coffee samples, Carbonell-Rozas et al. [43] examined ergot alkaloids in cereals, Bouti et al. [44] and Saber et al. [45] analyzed *Aspergillus* section Flavi strains in animal feed, Medjdoub et al. [46] reported contamination in couscous and peppers, Redouane-Salah et al. [47] detected aflatoxins in coffee, Jedidi et al. [48] studied AFM1 in various milk types, and Belasli et al. [49] assessed multiple mycotoxins in nuts, dried fruits, and cereal products. Laouni et al. [50] expanded research to emerging mycotoxins such as enniatins and beauvericin in poultry feed and eggs.

Parallel efforts focused on natural antifungal and detoxification approaches, which gained increasing attention. Badji et al. [10] explored the detoxification potential of lactic acid bacteria against AFB1 and OTA in wheat, Boudjaber et al. [51] evaluated *Chamaerops humilis* extracts as food preservatives, and Ben Miri et al. [11,52,53] conducted a series of studies on essential oils (*Mentha pulegium*, *Myrtus communis*, *Mentha piperita*, menthol, and eugenol) against *Aspergillus* species, demonstrating their potential as eco-friendly fumigants in couscous, stored grains, and coffee beans.

Complementary investigations addressed industrial and agricultural interventions. Belabed et al. [54] showed the efficacy of triazole fungicides against *Fusarium* species, Ait Issad et al. [55] and Mimoune et al. [56] demonstrated the benefits of mycotoxin binders and acidifiers in broiler farms, and Houari et al. [57] evaluated a feed additive improving milk production and udder health in dairy cattle.

Taken together, the defining characteristic of this phase was its broadening of research across food and feed matrices and stronger emphasis on natural and industrial control strategies, while the adoption of innovative detection methodologies remained limited.

A parallel and equally important development during this phase was the increasing role of Algerian researchers in international collaborations, which significantly amplified the scope and impact of national research. These partnerships, often involving European, North African, and American institutions, provided access to advanced chromatographic and molecular tools, facilitated participation in multi-centre studies, and enabled Algerian teams to contribute to large-scale assessments of mycotoxin risks. For example, Belasli et al. [49] collaborated with Spanish institutions to evaluate multi-mycotoxin contamination in nuts and cereals, integrating local sampling with advanced analytical methodologies. Badji et al. [10] worked with partners from Morocco, Tunisia, and France to identify lactic acid bacteria strains capable of detoxifying AFB1 and OTA in wheat products, highlighting the potential of biological mitigation strategies. Similarly, Laraba et al. [31] engaged in a multinational effort with Italy, the USA, and Australia to study the genetic structure and toxin production of *F. culmorum*, providing insights into both regional and global wheat safety challenges.

These examples demonstrate that while domestic research capacity expanded, international partnerships played a decisive role in strengthening methodological rigor, fostering knowledge exchange, and positioning Algerian researchers as active contributors to the global mycotoxin research community.

## 4. Mycotoxin Contamination in Algerian Agriculture and Food Supply

Algerian agricultural practices contribute to the risk of mycotoxin occurrence. The sector encompasses a wide array of crops, each cultivated using different methods. Traditional farming practices, such as minimal pesticide use and reliance on rain-fed agriculture, may heighten crop susceptibility to mycotoxin contamination. Furthermore, practices like crop rotation and soil management techniques influence the prevalence of mycotoxin-producing fungi in agricultural fields. Post-harvest handling and storage practices play a pivotal role in mycotoxin contamination levels in Algerian agricultural products. Inadequate storage facilities, combined with insufficient temperature and humidity control, can lead to mould growth and mycotoxin accumulation in stored grains, nuts, and other commodities.

Furthermore, Algerian dietary habits and consumption patterns also contribute to mycotoxin exposure. Staple foods and traditional dishes such as couscous, bread, and fermented dairy products may be vulnerable to mycotoxin contamination depending on production methods and ingredient quality. Studies have documented the presence of *Aspergillus* species and aflatoxin contamination in different date varieties cultivated across Algerian regions. Local regulations and monitoring initiatives have been developed to address this issue, although further research is required to strengthen prevention and control strategies. Table 1 outlines the occurrence of various mycotoxins in agricultural products and foodstuffs from Algeria.

In Algeria, the absence of stringent regulations and limited attention to the economic, health, and food security impacts of mycotoxin contamination exacerbate multiple challenges similar to those in other African nations. Economically, Algerian farmers endure significant losses when their crops are affected by mycotoxins, as inadequate market standards and regulatory frameworks often compel them to sell contaminated produce locally at reduced prices, further straining their livelihoods. Moreover, mycotoxin contamination seriously inhibits Algerian agricultural exports; non-compliance with international standards frequently leads to product rejections, resulting in considerable revenue losses.

This problem extends beyond Algeria’s borders, as African agricultural products commonly face high rejection rates in global markets due to mycotoxins. According to Chilaka et al. [7], the Rapid Alert System for Food and Feed (RASFF) attributes an average of 39% of EU annual border rejections of African food and feed products to mycotoxins. These rejections not only damage the exporting country’s reputation but also drive up export costs and reduce revenue due to the loss of products, as well as added expenses in logistics, transportation, and insurance. Between 2005 and 2020, the EU rejected 579 shipments of African agricultural products for exceeding legislative limits on mycotoxins, particularly aflatoxins, with Egypt, Nigeria, and South Africa among the most affected. Many African nations experienced rejections due to mycotoxins, especially AFB1 and total aflatoxins, in key exports such as groundnuts. For example, Egypt had a high number of rejections for groundnuts and unshelled groundnuts, with AFB1 concentrations reaching up to 11,000 µg/kg in some instances. Angola, in 2020, recorded a single rejection of groundnut kernels with AFB1 levels of 175 µg/kg. Other countries, including Ethiopia, Nigeria, Sudan, and South Africa, faced similar issues with groundnut and spice products, with aflatoxin levels varying significantly. Ethiopia’s ground berbere spice, for instance, recorded AFB1 levels up to 50.4 µg/kg. This data underscores the widespread non-compliance across African countries and highlights the pressing need for standardized mycotoxin regulations and improved contamination control to reduce export rejections and prevent economic losses [7].

Furthermore, mycotoxin-contaminated crops pose serious health risks to Algerian consumers. Inadequate regulations and oversight may allow contaminated food products to enter the market, exposing consumers to mycotoxins, causing liver damage, cancer, and other illnesses. This is exacerbated by a general lack of awareness about mycotoxin contamination, particularly among vulnerable groups such as children and pregnant women. Notably, the only study by Mendes et al. [61] shed light on the prevalence of mycotoxin exposure among Algerian workers, revealing the presence of multiple mycotoxins, including T-2, ZEN, and OTA. These findings highlight the urgent need for accurate data and enhanced regulatory measures to address mycotoxin contamination in Algeria, emphasizing the imperative of tackling this burgeoning public health concern.

Moreover, mycotoxin contamination undermines food security in Algeria by reducing the availability of safe and nutritious food. Unfit for human consumption, contaminated crops can lead to potential shortages, particularly in rural areas where agriculture is crucial for food production. Weak regulatory measures further compromise the integrity of the food supply chain, heightening the risk of widespread contamination and foodborne illnesses. Additionally, improper disposal of mycotoxin-contaminated crops can have adverse environmental consequences, with contaminated agricultural waste leaching mycotoxins into the soil, posing long-term risks to environmental and agricultural sustainability. To comprehensively tackle this issue, it is crucial to expand regulatory measures to cover a wider range of mycotoxins and include all locally consumed agricultural products, with strict adherence to set limits [62].

According to the FAO, only 28% of African countries had established mycotoxin regulations by 2003, with Morocco being the only country to update its standards in recent years [63]. This limited regulatory framework is concerning, as many nations apply these standards solely to export-bound products, neglecting domestic markets where mycotoxin exposure poses significant health risks. For instance, in Malawi, mycotoxin limits are enforced only for peanuts intended for export, despite the crop’s essential role in local diets [64]. Similarly, Côte d’Ivoire prioritizes monitoring economically valuable export crops like cocoa and coffee, limiting protections for the local population [65]. Such exclusive approaches often result in high-quality produce being reserved for international markets while domestic consumers are left exposed to potentially contaminated food. Nonetheless, this gap could catalyze strategic improvements in agricultural practices to reduce fungal contamination in local food products. Detailed Algeria-specific data are provided in Table S1 (Supplementary Materials).

In contrast, Morocco has implemented one of the most comprehensive and current mycotoxin regulatory frameworks on the continent, aligning its standards with those of the European Union (EU). These regulations cover aflatoxins, fumonisins, ochratoxin A, deoxynivalenone, patulin, and zearalenone across various food products [63]. However, despite pioneering research on fumonisins and frequent reports of mycotoxin occurrences, South Africa still lacks formal regulatory limits for these toxins [66]. Nigeria has established maximum levels for total aflatoxins in products like maize, raw groundnuts, groundnut cake, sesame seeds, and sorghum, with limits at 10, 20, 4, 4, and 10 μg/kg, respectively [67]. These standards largely reflect EU regulations but are primarily aimed at meeting trade requirements rather than ensuring local food safety.

Globally, the European Commission (EC) and the United States Food and Drug Administration (FDA) have established maximum levels for several mycotoxins in food. For instance, EC guidelines (2023) limit AFB1 in cereals to 2–4 µg/kg and 2-12 μg/kg in dried fruits and nuts, with specific thresholds for AFB1 and total AFs across multiple food categories. Limits are also set for groundnuts, almonds, pistachios, apricot kernels, cereals, and rice, capping AFB1 at 2-5 μg/kg and total AFs at 4-10 μg/kg. Dried spices, including ginger, have an AFB1 limit of 5.0 μg/kg and a total AF threshold of 10.0 μg/kg. In dairy, AFM1 is strictly limited to 0.050 μg/kg in milk and 0.025 μg/kg in infant formula. Additional MLs for mycotoxins like FBs and OTA range from 200 to 1,000 μg/kg for cereals and cereal products, and from 2 to 10 μg/kg for wine, coffee, cheese, and cocoa, with limits for ZEA, DON, and PAT also specified across cereals and beverages [62,68].

In Algeria, current regulations establish a maximum limit of 10 µg/kg for AFB1 and 20 µg/kg for total aflatoxins (B1, B2, G1, and G2), in peanuts, nuts, and cereals [69,70]. These standards protect public health and facilitate compliance with international requirements. However, beyond aflatoxins, numerous other mycotoxins and important foodstuffs in the region remain unregulated, leaving significant gaps in consumer protection. Comparing Algeria’s regulatory framework to global benchmarks highlights opportunities for adopting best practices from organizations like the Codex Alimentarius Commission and the FDA, thereby enhancing Algeria’s alignment with international norms. Recent analyses show instances where mycotoxin levels in Algerian products exceed EU thresholds, underscoring the need for stringent monitoring and regulatory practices to protect public health [47].

Research on fungal contamination and mycotoxins in dates highlights both scientific advances and persistent gaps. Although regulatory frameworks and monitoring systems have been implemented in several producing countries, challenges remain in standardizing methodologies, ensuring continuous surveillance, and integrating preventive measures across the supply chain. Future studies should emphasize sustainable control strategies, such as bioactive edible coatings, biocontrol agents, and predictive modeling, while also considering consumer safety and international trade requirements.

## 5. Methods and Challenges in Mycotoxin Analysis

The detection of mycotoxins plays a pivotal role in safeguarding public health due to their toxicity even at trace levels. Consequently, employing sensitive and reliable techniques is imperative. Accurate assessment of mycotoxin levels enables the implementation of effective mitigation strategies, thereby reducing the risk associated with consuming highly contaminated food and ensuring compliance with regulatory standards set by entities like the United States of America, the European Union, and other international organizations [71].

Efficient and cost-effective screening and analytical methods are essential for mycotoxin detection in Algeria. Chromatographic separations, especially when coupled with mass spectrometry, provide precise identification of mycotoxins. Various techniques, including HPLC, UHPLC-MS/MS, LC-MS/MS, TLC, and ELISA, are used for mycotoxin detection by Algerian researchers [28,31,47,59,62].

Furthermore, Algerian researchers face specific challenges and limitations in mycotoxin analysis, ranging from sample preparation intricacies to issues of sensitivity and validation. Sample preparation methods stand as the cornerstone of reliable analysis, yet their optimization poses a significant challenge. Researchers must navigate matrix effects and analyte stability, ensuring that the preparation process does not compromise the integrity of the sample or skew results. Moreover, the request for sensitivity adds another layer of complexity. Detecting mycotoxins at trace levels demands a level of precision that leaves no room for error. Even minor variations in analytical parameters can have profound implications for the outcome, underscoring the need for attention to detail.

Validation of analytical protocols is also vital to ensure reproducibility and data reliability. This requires the implementation of robust quality assurance measures at every stage of the analytical process, from method development to sample analysis. Nevertheless, only a limited number of Algerian studies report full validation parameters (LOD, LOQ, recovery rates), which is critical to ensure data comparability and compliance with EU standards.

However, beyond the technical intricacies, Algerian researchers face the stark reality of limited access to cutting-edge techniques and standards. Often, these methodologies are either unavailable or inaccessible, posing a significant barrier to progress. Even when the techniques themselves are within reach, the absence of standardized protocols or certified standards further complicates matters, hindering the attainment of accurate and reliable results. However, the implementation of advanced detection techniques in Algeria is hindered by resource constraints, including limited access to high-end equipment and funding. Many analytical analyses are conducted through collaborations with foreign institutions, underscoring the importance of international partnerships in enhancing Algeria’s mycotoxin detection capabilities. To address these challenges, investment in domestic infrastructure and capacity-building programs is essential. Fostering the development of local expertise and research capabilities can enhance Algeria’s capacity to detect and manage mycotoxin contamination more effectively in the long term. Collaborations with foreign institutions provide valuable support, but efforts to build domestic capacity are equally critical.

## 6. Bridging the Gap: Future Research Directions

Mycotoxin research in Algeria is marked by significant accomplishments, yet intertwined with persistent challenges. As scientists continue to investigate the complex dynamics of mycotoxin occurrence, detection, and their implications for food safety and public health, they often confront obstacles such as limited resources and the need for tailored, context-specific solutions. Despite these challenges, Algerian researchers have shown notable resilience and creativity, laying the groundwork for future advances in the field. Through the exploration of native remedies, strengthening surveillance systems, and fostering interdisciplinary collaboration, Algeria not only has the potential to overcome current barriers but also to wield considerable global influence in addressing mycotoxin contamination:

(a) Given the limited application of advanced technologies in mycotoxin detection, there is growing interest in exploring traditional Algerian food preservation practices and natural compounds believed to mitigate mycotoxin contamination. Researchers are investigating time-honored techniques passed down through generations, aiming to uncover culturally relevant and sustainable strategies.
(b) Building resilient surveillance systems involves implementing simple yet effective methods to monitor mycotoxin levels in Algerian agricultural products and regions. This proactive approach allows for the early identification of areas with high contamination risks, enabling prompt intervention measures despite technological limitations.
(c) Algerian researchers foster interdisciplinary teamwork among experts from agriculture, food science, microbiology, and public health to develop comprehensive solutions to mycotoxin contamination. Leveraging diverse expertise enables a deeper understanding of contamination factors, facilitating holistic approaches despite limited technological advancements.
(d) Collaboration with international institutions, industry stakeholders, and governmental organizations remains crucial for accessing funding, expertise, and advanced technologies. Sharing resources and exchanging knowledge helps overcome limitations posed by scarce resources, accelerating research and development efforts in mycotoxin mitigation.
(e) Implementing training programs and workshops is essential for enhancing the skills and knowledge of local researchers, extension workers, and food industry professionals. Investing in human capital strengthens Algeria’s research infrastructure and establishes a sustainable framework for addressing mycotoxin contamination in the absence of advanced technology.
(f) Conducting thorough risk assessments remains vital for identifying situations with high mycotoxin exposure risks and vulnerable population groups despite technological limitations. Developing targeted management strategies, such as promoting traditional agricultural practices and implementing regulatory measures, is essential for effectively mitigating risks and ensuring compliance with food safety standards.

Altogether, these directions underscore the potential of Algeria to not only strengthen its domestic food safety framework but also contribute meaningfully to the global dialogue on mycotoxin mitigation.

## 7. Conclusions

The comprehensive study of mycotoxin research and publications in Algeria emphasizes the significant strides in understanding mycotoxin occurrence, detection techniques, and their impact on food safety. Despite facing resource limitations such as access to advanced technology, Algerian researchers have shown resilience and innovation in their approaches. Although the scientific literature on mycotoxins in Algeria is still relatively scarce, each publication contributes valuable insights into various aspects of mycotoxin contamination in Algerian agricultural products and foodstuffs. These contributions, while limited in number, form the foundation for improving management strategies and developing context-specific solutions.

Algeria’s diverse environmental conditions offer unique opportunities for studying mycotoxin dynamics, and by prioritizing localized solutions, researchers can devise interventions applicable not only within Algeria but also in analogous regions worldwide. Collaborative efforts, including interdisciplinary methodologies and partnerships with international collaborators, further enrich the landscape of mycotoxin research in Algeria and pave the way for potential global impact.

## Figures and Tables

**Table 1:** Mycotoxin occurrence in food and feed products from Algeria.

Mycotoxins	Agricultural Commodity	Concentration	N (Samples)	Reference
OTA	Wheat	0.21–27.31 μg/kg	39	[9]
OTA	Semolina	0.16–34.75 μg/kg	29	[9]
OTA	Flour	0.16–34.75 μg/kg	13	[9]
DON	Wheat	5000–15000 µg/kg	70	[32]
FB1	Maize	289–42.143 μg/kg	30	[37] ^3^
FB2	Maize	27.5–8603 μg/kg	30	[37] ^3^
T–2	Maize	24.6–25.7 μg/kg	30	[37] ^3^
DON	Maize	47.6–2055 μg/kg	30	[37] ^3^
ZEN	Maize	20.4–579 μg/kg	30	[37] ^3^
F–X	Maize	177–477 μg/kg	30	[37] ^3^
CIT	Maize	8.6–273 μg/kg	30	[37] ^3^
BEA	Maize	0.85–31.4 μg/kg	30	[37] ^3^
ENNA1	Maize	11.5–103 μg/kg	30	[37] ^3^
ENNB1	Maize	15.0–107 μg/kg	30	[37] ^3^
HT–2	Wheat	8.4–36.7 μg/kg	30	[37] ^3^
DON	Wheat	68.3–1363 μg/kg	30	[37] ^3^
ZEN	Wheat	9.6–295 μg/kg	30	[37] ^3^
F–X	Wheat	139–159 μg/kg	30	[37] ^3^
OTA	Wheat	20–92 μg/kg	30	[37] ^3^
CIT	Wheat	9.8–32.3 μg/kg	30	[37] ^3^
STE	Wheat	0.6–1.3 μg/kg	30	[37] ^3^
BEA	Wheat	2.8–486 μg/kg	30	[37] ^3^
ENNA	Wheat	8.4–87.6 μg/kg	30	[37] ^3^
ENNA1	Wheat	4.0–395 μg/kg	30	[37] ^3^
ENNB	Wheat	1.2–5288 μg/kg	30	[37] ^3^
ENNB1	Wheat	19.5–4569 μg/kg	30	[37] ^3^
Ergot alcaloids	Wheat	3.66–76.0 μg/kg	41	[43]
Ergot alcaloids	Barley	17.8–53.9 μg/kg.	43	[43]
AFB1	Wheat grain stored in silo ^1^	0.13–37.42 μg/kg	108	[18]
AFB1	Pre-harvest wheat grain ^2^	0.21-13.96 μg/kg	108	[18]
AFB1	Semolina	1.18 μg/kg	108	[18]
AFB1	Bran	3.37 μg/kg	108	[18]
OTA	Wheat grain stored in silo ^1^	0.21- 3.91 μg/kg	30	[17]
OTA	Pre-harvest wheat grain ^2^	0.45–1.65 μg/kg	30	[17]
AFB1	Roasted hazelnuts	0.20–2.81 μg/kg	88	[21]
AFB1	Shelled almonds	1.65–4.00 μg/kg	8	[21]
AFB1	Shelled peanuts	0.34–25.82 μg/kg	8	[21]
AFB1	Pistachio	0.28–8.72 μg/kg	8	[21]
AFB1	Unshelled walnuts	0.20–6.34 μg/kg	8	[21]
AFs	Pistachios	0.4–0.7 μg/kg	31	[58]
OTA	Pistachios	170 μg/kg	31	[58]
OTA	Cereal-based products	0.15 μg/kg	71	[49]
DON	Cereal-based products	90–123 μg/kg	71	[49]
AFs	Dried figs, dates, and bradj pastries	0.03–0.49 μg/kg.	62	[49]
AFM1	Raw milk	9–103 ng/L	47	[22]
AFM1	Powdered milk	20.34 ng/L	13	[48]
AFM1	Cow milk	5.8 ng/L	21	[48]
AFB1	Spices	0.10–26.50 μg/kg 0.10-25.82 μg/kg	44	[27]
AFs	Couscous	21.75 μg/kg	4	[46]
OTA	Grapes	<30 ng/L	30	[36]
ENNB1	Poultry feed, eggs	3.6–41.5 μg/kg	35	[50]
BEA	Poultry feed	12 μg/kg	10	[50]
AFs	Coffee	1.004-1.167 μg/kg	43	[47]
AFs	Animal feed	0.34-171.06 μg/kg	101	[44]
FB1	Poultry feed	<400–>3000 μg/kg	69	[59]
OTA	Poultry feed	0.02–63 μg/kg	*n.r.*	[60]
OTA	Poultry organs	570–9730 ng/L	*n.r*.	[60]
DON	Wheat	5000–15000 µg/kg	70	[32]
AFM1	Raw cow milk	95.59–557.22 ng/L	84	[38]
AFs	Peanuts	0.71–25.50 μg/kg	50	[20]

AFB1 = aflatoxin B1; AFG1 = aflatoxin G1; AFM1 = aflatoxin M1; AFs = total aflatoxins; ENN = Enniatins; OTA = ochratoxin A; FB1 = fumonisin B1; FB2 = fumonisin B2; FB3 = fumonisin B3; ZEA = zearalenone; DON = deoxynivalenol; ENN = enniatin; BEA = beauvericin; CIT = citrinin; STE = sterigmatocistin; T-2 = T-2 toxin. F–X= fusarenon X; ^1^ Wheat variety *Waha* and *Vitron* stored in silos for six months. ^2^ Pre-harvest variety *Waha.* ^3^ All mycotoxins listed under reference [37] derive from the same survey including multiple matrices. *n.r. =* not reported.

## Data Availability

No new data were created or analyzed in this study.

## Supplementary Materials

The following supporting information can be downloaded at: https://www.mdpi.com/article/10.3390/toxins17100492/s1, Table S1. Mycotoxins regulatory limits in foodstuffs in Africa.
