# Supplementary material for: Mycotoxin Research in Algeria: A Comprehensive Review of Trends, Challenges, and Future Directions
Source: Toxins (Basel). 2025 Oct 3;17(10):492. doi: 10.3390/toxins17100492 (PMC12568260; doi:10.3390/toxins17100492)
Supplement: Supplementary file 1 [file toxins-17-00492-s001.zip › toxins-3876535-supplementary.pdf]

Supplementary Materials: Mycotoxin Research in Algeria: A Comprehensive Review of Trends, Challenges, and Future Directions

Yamina Ben Miri, Imene Chentir, Aldjia Taoudiat, Amina Benabdallah, Marta Herrera

Table S1. Mycotoxins regulatory limits in foodstuffs in Africa.

Country	Mycotoxin Type	Commodity	Maximum Limits (µg/kg)	Reference
Algeria	AFB1	Peanuts, nuts, cereals	10	[69]
	AFB1, B2, G1, G2	Peanuts, nuts, cereals	20	
Egypt	AFB1	Peanuts and cereals	5	[69]
	AFB1, B2, G1, G2	Peanuts and cereals	10	
		Maize	10	
	AFB1	Maize	20	
	AFB1, B2, G1, G2	Milk intended for adults	0.5	
	AFM1	Coffee	5	
	OTA	Wheat and wheat flour	700	
	DON	Barley and barley flour	1000	
Morocco	AFB1	Peanuts and other oil seeds, hazelnuts and walnuts intended for sorting or other physical methods before human consumption or use as an ingredient in food products, unless they are intended to be crushed for the manufacture of refined vegetable oil.	8	[63]
	AFB1, B2, G1, G2		15	
			12	
	AFB1		15	
	AFB1, B2, G1, G2	Almonds, pistachios and apricot kernels		

AFB1<br>AFB1, B2, G1, G2	intended for sorting or other physical methods before human consumption or use as food ingredients.	5<br>10
AFB1<br>AFB1, B2, G1, G2	Other nuts (except nuts listed in 1 and 2) intended for sorting or other physical methods before human consumption or use as food ingredient.	2<br>4
AFB1<br>AFB1, B2, G1, G2	Peanuts and other oil seeds and their products intended for direct human consumption or use as ingredients for food products, with the exception of crude vegetable oils intended to be refined and refined vegetable oils.	8<br>10
AFB1<br>AFB1, B2, G1, G2	Almonds, pistachios and apricot kernels intended for direct human consumption or use as food ingredients.	5<br>10<br>2<br>4
AFB1<br>AFB1, B2, G1, G2	Hazelnuts and Brazil nuts for direct human consumption or use as an ingredient in foodstuffs.	

AFB1<br>AFB1, B2, G1, G2	Nuts (except nuts listed in 5 and 6) and their products intended for direct human consumption or for direct use as an ingredient in foodstuffs	5<br>10<br>2
AFB1<br>AFB1, B2, G1, G2	Dried fruits, other than dried figs intended for sorting or other physical methods before human consumption or use as food ingredients	4<br>6<br>10
AFB1<br>AFB1, B2, G1, G2	Dried fruits and their products (other than dried figs) intended for direct human consumption or use as food ingredients.	5<br>10
AFB1<br>AFB1, B2, G1, G2	Dried fruits.	0.05 *
AFM1	Maize and rice intended for sorting or other physical methods before human consumption or use as an ingredient for food products.	0.025<br>5<br>10 *<br>5
	Raw milk, heat-treated milk and milk-based products	10
OTA		2 *

	Infant formulas including infant milk	
	Raw cereals	
	Raisins (currants, sultanas and others raisins)	0.5
	Roasted coffee beans including ground, except soluble coffee.	8
	Instant coffee (instant coffee).	
	Grape juice, grape must, reconstituted concentrated grape juice and grape must, intended for direct human consumption.	50 *<br>50 *
PAT	Cereal-based baby food intended for infants and young children.	10
	Wheat gluten not sold directly to the consumer.	
	Fruit juices, reconstituted fruit juice concentrates and fruit nectars.	
	Spirits, cider and other fermented drinks produced from apples or containing apple juice.	
	Foods for babies, other than cereal-based	

		products intended for infants and children.		
	DON	Raw cereals other than durum wheat, oats, rice and maize.<br>Durum wheat and raw oats.<br>Dry pasta.<br>Cereal-based baby food intended for infants and young children.	1250 *<br>1750 *<br>750 *<br>200	
	ZEN	Raw cereals other than corn and rice.<br>Refined corn oil.<br>Maize intended for direct human consumption, corn snacks and breakfast cereals.<br>Cereal-based baby food intended for infants and young children.	100 *<br>400 *<br>100<br>20	
	FB (sum B1 + B2)	Corn-based breakfast cereals and snacks corn-based.<br>Corn preparations and baby food intended for infants and young children.	800<br>200	
Mozambique	AFB1, B2, G1, G2	Peanut, peanut milk, peanut butter, maize,	10	[69]

		cereals and feedstuffs		
Nigeria	AFB1<br>AFB1, B2, G1, G2<br>AFB1, B2, G1, G2<br>AFB1, B2, G1, G2<br>AFB1, B2, G1, G2<br>AFM1	Maize<br>Maize<br>Sorghum, millet grains<br>Kuli kuli (groundnut cake), sesame seed, fruits and fruit products, baby and infant foods, tea, coffee and cocoa products, malt drink, wheat flour, composite flour, wheat semolina, shea butter and shea nut kernels.<br>Raw groundnut<br>Baby and infant foods.	2<br>4<br>10<br>4<br>20<br>0.05	[67,68]
South Africa	AFB1<br>AFB1, B2, G1, G2<br>PAT<br>AFM1	All foods<br>All foods<br>All foods<br>Milk and milk products.	5<br>10<br>50<br>0.05	[69]
Sudan	AFB1, B2, G1, G2<br>OTA	Oil seeds.<br>Wheat.	15<br>15	[69]
Tanzania	AFB1<br>AFB1, B2, G1, G2	Cereals, oil seeds.	5<br>10	[69,30]
Tunisia	AFB1	All products	2	[69]
Uganda	AFB1, B2, G1, G2	All foods.	10	[70]
Zimbabwe	AFB1<br>AFB1<br>AFG1	All foods.<br>Groundnuts, maize, sorghum.<br>Groundnuts, maize, sorghum.	5<br>5<br>4	[69]
